# Supplementary material for: Inequalities in the identification and management of common mental disorders in the perinatal period: An equity focused re-analysis of a systematic review
Source: PLoS One. 2021 Mar 15;16(3):e0248631. doi: 10.1371/journal.pone.0248631 (PMC7959342; doi:10.1371/journal.pone.0248631)
Supplement: S1 Table — (DOCX) [file pone.0248631.s002.docx]

**Inequalities in the identification and management of common mental disorders in the perinatal period: an equity focused re-analysis of a systematic review**

**S1 Table. Absolute and relative differences in case-finding equity**

|  |  |  | **Equity Estimates** | | | | |
| --- | --- | --- | --- | --- | --- | --- | --- |
| **Study** | **Measure** | **Whole sample estimate % (n/N)** | **Group** | **Within group estimate % (n/N)** | **Absolute % difference** | **No. of women per 100 [detected] in reference group** | **Relative Risk (95% CI)** |
| Redshaw & Henderson (2016) [38] | Recalled being asked about mental health (AN)* | 82.0 (3,597/4,428) | White  Mixed  Asian  Black  Other | 82.6 (3,045/3,715)  83.9 (73/87)  75.7 (331/444)  83.9 (130/159)  78.3 (18/23) | Reference  -1.3  6.9  -1.3  4.3 | Reference  101  93  101  96 | Reference  0.98  1.09  0.98  1.06 |
|  |  | 82.0 (3,641/4,484) | Left full time education 19+ years old  <17  17-18  Still in education | 82.5 (2,057/2,513)  80.6 (601/757)  81.7 (980/1,211)  100 (3/3) | Reference  1.9  0.8  -18 | Reference  98  99  118 | Reference  1.02  1.01  0.83 |
|  |  | 81.9 (3,704/4,570) | 1- least deprived quintile of IMD  2  3  4  5 - most deprived | 80.2 (716/899)  82.9 (708/865)  82.3 (763/935)  83.3 (803/977)  80.8 (714/894) | Reference  -2.7  -2.1  -3.1  -0.6 | Reference  103  102  103  101 | Reference  0.97  0.97  0.96  0.99 |
|  |  | 81.9 (3,593/4,430) | Primaparous  Multiparous | 83.1 (1,819/2,207)  80.7 (1,774/2,223) | Reference  2.4 | Reference  98 | Reference  1.03 |
|  | Recalled being asked about past or family history of mental health problems (AN)* | 84.1 (3,652/4,428) | White  Mixed  Asian  Black  Other | 84.9 (3,098/3,715)  83.7 (72/87)  79.1 (341/444)  81.0 (124/159)  77.3 (17/23) | Reference  1.2  5.8  3.9  7.6 | Reference  99  94  96  92 | Reference  1.01  1.07  1.05  1.10 |
|  |  | 84.1 (3,700/4,484) | Left full time education 19+ years old  <17  17-18  Still in education | 84.3 (2,083/2,513)  82.4 (607/757)  84.9 (1,007/1,211)  100 (3/3) | Reference  1.9  -0.6  -- | Reference  98  101  -- | Reference  1.02  0.99  -- |
|  |  | 84.0 (3,759/4,570) | 1- least deprived quintile of IMD  2  3  4  5 - most deprived | 81.5 (723/899)  84.7 (724/865)  84.5 (774/935)  85.1 (811/977)  84.3 (727/894) | Reference  -3.2  -3.0  -3.6  -2.8 | Reference  103  103  104  103 | Reference  0.96  0.96  0.96  0.97 |
|  |  | 84.2 (3,654/4,430) | Primaparous  Multiparous | 85.5 (1,856/2,207)  82.9 (1,798/2,223) | Reference  2.6 | Reference  97 | Reference  1.03 |
|  | Recalled being asked about mental health (PN) | 90.1 (3,963/4,400) | White  Mixed  Asian  Black  Other | 91.9 (3,399/3,698)  85.1 (74/87)  80.1 (350/437)  80.0 (124/155)  69.6 (16/23) | Reference  6.9  11.8  11.9  22.3 | Reference  93  88  88  78 | Reference  1.08 (0.99, 1.18)  **1.15 (1.09, 1.20)**  **1.15 (1.06, 1.24)**  **1.32 (1.01, 1.73)** |
|  |  | 90.0 (4,009/4,455) | Left full time education 19+ years old  <17  17-18 | 91.6 (2,286/2,495)  88.0 (662/752)  87.8 (1058/1,205) | Reference  3.6  3.8 | Reference  96  96 | Reference  **1.04 (1.01, 1.07)**  **1.04 (1.02, 1.07)** |
|  |  | 89.8 (4,044/4,501) | 1- least deprived quintile of IMD  2  3  4  5 - most deprived | 92.4 (825/893)  91.8 (787/857)  91.7 (844/920)  89.2 (859/963)  84.0 (729/868) | Reference  0.6  0.6  3.2  8.4 | Reference  99  99  97  92 | Reference  1.01 (0.98, 1.03)  1.01 (0.98, 1.03)  **1.04 (1.01, 1.07)**  **1.10 (1.06, 1.14)** |
|  |  | 89.9 (3,958/4,402) | Primaparous  Multiparous | 91.2 (2,000/2,194)  88.7 (1,958/2,208) | Reference  2.5 | Reference  98 | Reference  **1.03 (1.01, 1.05)** |
| Prady et. al. (2016) [22] | GP record of case finding activity (AN, pregnancy) | 1.7 (153/8,991) | White British  Pakistani (English)  Other (English)  Any (not English) | 2.7 (95/3,546)  1.3 (33/2,602)  1.2 (15/1,209)  0.6 (10/1,634) | Reference  1.4  1.4  2.1 | Reference  99  99  98 | Reference  **2.11 (1.43, 3.13)**  **2.16 (1.27, 3.75)**  **4.38 (2.29, 8.38)** |
|  | Potentially missed CMD (AN, pregnancy and pre-pregnancy) (at GHQ-28 ≥15)** | 31.3 (251/1,168) | White British  Pakistani (English)  Other (English)  Any (not English) | 17.1 (424/536)  45.3 (196/405)  44.1 (64/129)  39.8 (53/98) | Reference  37.7  38.8  43.0 | Reference  62  61  57 | Reference  **2.66 (2.17, 3.26)**  **2.59 (2.00, 3.35)**  **2.33 (1.74, 3.14)** |
|  | GP record of case finding activity (PN, up to 1 year) | 12.7 (1,141/8,991) | White British  Pakistani (English)  Other (English)  Any (not English) | 18.0 (640/3,546)  10.1 (263/2,602)  10.7 (129/1,209)  6.7 (109/1,634) | Reference  7.9  7.4  11.4 | Reference  92  93  89 | Reference  **1.79 (1.56, 2.04)**  **1.69 (1.41, 2.02)**  **2.71 (2.23, 3.29)** |

* There is a mismatch between the reported n/N and percentages; we have presented the n/N as reported in the paper in order to indicate the size of each group and present the percentages reported, but have not calculated the confidence intervals around the relative risk due to uncertainty about precision. ** Percentages and relative estimates are weighted, they are presented as reported in the paper and not calculated here (unweighted n’s are provided to indicate the sample size, but will not sum to the weighted percentages). GHQ-28 ≥15, the threshold of the GHQ-28 above which the women was classified as potentially having a CMD. A negative absolute difference indicates a higher level of detection than the reference group. Relative risk is expressed as >1 indicating a lower level of detection for the disadvantaged group.Bolded estimates indicate a statistically significant difference, and are only indicated where confidence intervals could be constructed. Comparative statistics only computed where the denominator was greater than 5.
