# Supplementary material for: Inequalities in the identification and management of common mental disorders in the perinatal period: An equity focused re-analysis of a systematic review
Source: PLoS One. 2021 Mar 15;16(3):e0248631. doi: 10.1371/journal.pone.0248631 (PMC7959342; doi:10.1371/journal.pone.0248631)
Supplement: S2 Table — (DOCX) [file pone.0248631.s003.docx]

**Inequalities in the identification and management of common mental disorders in the perinatal period: an equity focused re-analysis of a systematic review**

**S2 Table. Absolute and relative differences in management equity (Redshaw and Henderson, 2016) [38]**

|  |  |  | **Equity Estimates** | | | |
| --- | --- | --- | --- | --- | --- | --- |
| **Measure** | **Whole sample estimate % (n/N)** | **Group** | **Within group estimate % (n/N)** | **Absolute % difference** | **No. of women per 100 [treated] in reference group** | **Relative Risk (95% CI)** |
| Offered treatment (AN) | 36.6 (325/888) | White  Mixed  Asian  Black  Other | 41.2 (277/672)  40.9 (9/22)  19.6 (29/148)  17.9 (7/39)  37.5 (3/8) | Reference  0.30  21.6  23.3  3.7 | Reference  100  78  77  96 | Reference  1.01 (0.61, 1.68)  **2.10 (1.50, 2.95)**  **2.30 (1.17, 4.52)**  1.10 (0.45, 2.70) |
|  | 36.1 (329/911) | Left full time education 19+ years old  <17  17-18  Still in education | 32.3 (144/446)  39.7 (83/209)  39.9 (101/253)  50.0 (1/2) | Reference  -7.4  -7.6  -- | Reference  107  108  -- | Reference  0.81 (0.66, 1.01)  **0.81 (0.66, 0.99)**  -- |
|  | 35.6 (336/944) | 1- least deprived quintile of IMD  2  3  4  5 - most deprived | 40.2 (49/122)  35.1 (46/131)  39.0 (69/177)  33.2 (75/226)  33.6 (97/289) | Reference  5.1  1.2  7.0  6.6 | Reference  95  99  93  93 | Reference  1.15 (0.83, 1.57)  1.03 (0.78 1.37)  1.21 (0.91, 1.61)  1.20 (0.91, 1.57) |
|  | 35.7(318/891) | Primaparous  Multiparous | 32.9 (136/413)  38.2 (182/476) | Reference  -5.3 | Reference  105 | Reference  0.86 (0.72, 1.50) |
| Received support (AN) | 70.4 (207/294) | White  Mixed  Asian  Black  Other | 74.4 (177/238)  50.0 (4/8)  52.8 (19/36)  50.0 (5/10)  100 (2/2) | Reference  24.4  21.6  24.4  -- | Reference  76  78  76  -- | Reference  1.49 (0.74, 2.99)  **1.41 (1.03, 1.94)**  1.49 (0.80, 2.78)  **--** |
|  | 69.2 (211/305) | Left full time education 19+ years old  <17  17-18 | 74.5 (108/145)  57.7 (45/78)  70.7 (58/82) | Reference  16.8  3.8 | Reference  83  96 | Reference  **1.29 (1.04, 1.60)**  1.05 (0.89, 1.25) |
|  | 68.3 (213/312) | 1- least deprived quintile of IMD  2  3  4  5 - most deprived | 80.6 (29/36)  77.8 (35/45)  75.0 (48/64)  61.0 (47/77)  60.0 (54/90) | Reference  2.8  5.6  19.6  20.6 | Reference  97  94  80  79 | Reference  1.04 (0.83, 1.30)  1.07 (0.87, 1.33)  **1.32 (1.04, 1.68)**  **1.34 (1.06, 1.70)** |
|  | 68.4 (214/313) | Primaparous  Multiparous | 71.2 (166/233)  60.0 (48/80) | Reference  11.2 | Reference  89 | Reference  1.19 (0.98, 1.45) |
| Received advice (AN) | 73.5 (219/298) | White  Mixed  Asian  Black  Other | 76.4 (188/246)  57.1 (4/7)  55.6 (20/36)  71.4 (5/7)  100 (2/2) | Reference  19.3  20.8  5.0  -- | Reference  81  79  95  -- | Reference  **1.34 (0.70, 2.55)**  **1.37 (1.02, 1.86)**  1.07, (0.67, 1.72)  -- |
|  | 71.9 (225/313) | Left full time education 19+ years old  <17  17-18 | 73.0 (108/148)  64.6 (51/79)  76.7 (66/86) | Reference  8.4  -3.7 | Reference  92  104 | Reference  1.13 (0.93, 1.37)  0.95 (0.82, 1.11) |
|  | 71.3 (228/320) | 1- least deprived quintile of IMD  2  3  4  5 - most deprived | 80.5 (33/41)  76.2 (32/42)  72.3 (47/65)  72.5 (58/80)  63.0 (58/92) | Reference  4.3  8.2  8.0  17.5 | Reference  96  92  92  83 | Reference  1.06 (0.84, 1.32)  1.11 (0.90, 1.40)  1.11 (0.91, 1.36)  **1.28 (1.03, 1.59)** |
|  | 71.3 (220/309) | Primaparous  Multiparous | 67.9 (91/134)  74.1 (129/174) | Reference  -6.2 | Reference  106 | Reference  0.92(0.79, 1.06) |
| Received treatment (AN) | 47.3 (115/243) | White  Mixed  Asian  Black  Other | 46.2 (92/199)  60.0 (3/5)  46.7 (14/30)  62.5 (5/8)  100 (1/1) | Reference  --  -0.5  -16.3  -- | Reference  --  101  116  -- | Reference  --  0.99 (0.65, 1.49)  0.74 (0.42, 1.29)  -- |
|  | 45.1 (115/255) | Left full time education 19+ years old  <17  17-18 | 48.3 (56/116)  44.9 (31/69)  40.0 (28/70) | Reference  3.4  8.3 | Reference  97  92 | Reference  1.08 (0.78, 1.48)  1.21 (0.86, 1.70) |
|  | 45.0 (118/262) | 1- least deprived quintile of IMD  2  3  4  5 - most deprived | 46.9 (15/32)  32.4 (11/34)  48.0 (24/50)  43.1 (28/65)  49.4 (40/81) | Reference  14.5  -1.1  3.8  -2.5 | Reference  86  101  96  103 | Reference  1.45 (0.79, 2.67)  0.98 (0.61, 1.56)  1.09 (0.69, 1.73)  0.95 (0.62, 1.46) |
|  | 44.4 (110/248) | Primaparous  Multiparous | 40.7 (44/108)  47.1 (66/140) | Reference  -6.4 | Reference  106 | Reference  0.86 (0.65, 1.15) |
| Received support (PN) | 63.7 (258/405) | White  Mixed  Asian  Black  Other | 67.4 (217/322)  90.0 (9/10)  42.9 (24/56)  46.2 (6/13)  50.0 (2/4) | Reference  -22.6  24.5  21.2  -- | Reference  123  76  79  -- | Reference  **0.75 (0.61, 0.93)**  **1.57 (1.15, 2.15)**  1.46 (0.81, 2.64)  -- |
|  | 63.4 (260/410) | Left full time education 19+ years old  <17  17-18 | 62.6 (132/211)  68.5 (61/89)  60.9 (67/110) | Reference  -5.9  1.7 | Reference  106  98 | Reference  0.91 (0.77, 1.09)  1.03 (0.86, 1.23) |
|  | 63.2 (263/416) | 1- least deprived quintile of IMD  2  3  4  5 - most deprived | 70.4 (50/71)  66.7 (40/60)  69.0 (60/87)  60.0 (57/95)  54.4 (56/103) | Reference  3.7  1.4  10.4  16.0 | Reference  96  99  90  84 | Reference  1.06 (0.84, 1.33)  1.02 (0.83, 1.26)  1.17 (0.94, 1.47)  **1.29 (1.03, 1.63)** |
|  | 63.4 (253/399) | Primaparous  Multiparous | 61.1 (110/180)  65.3 (143/219) | Reference  -4.2 | Reference  104 | Reference  0.94 (0.80, 1.09) |
| Received advice (PN) | 64.4 (262/407) | White  Mixed  Asian  Black  Other | 67.3 (216/321)  70.0 (7/10)  50.8 (31/61)  58.3 (7/12)  33.3 (1/3) | Reference  -2.7  16.5  9  -- | Reference  103  84  91  -- | Reference  0.96 (0.64, 1.45)  **1.32 (1.02, 1.71)**  1.15 (0.71, 1.87)  -- |
|  | 63.9 (264/413) | Left full time education 19+ years old  <17  17-18 | 61.6 (133/216)  71.9 (64/89)  62.0 (67/108) | Reference  -10.3  -0.4 | Reference  110  100 | Reference  0.86 (0.72, 1.01)  0.99 (0.83, 1.19) |
|  | 63.5 (266/419) | 1- least deprived quintile of IMD  2  3  4  5 - most deprived | 69.6 (48/69)  61.7 (37/60)  72.1 (62/86)  61.1 (58/95)  56.0 (61/109) | Reference  7.9  -2.5  8.5  13.6 | Reference  92  103  92  86 | Reference  1.13 (0.88, 1.45)  0.97 (0.79, 1.18)  1.14 (0.91, 1.43)  1.24 (0.99, 1.56) |
|  | 63.4 (255/402) | Primaparous  Multiparous | 61.2 (112/183)  65.3 (143/219) | Reference  -4.1 | Reference  104 | Reference  0.94 (0.81, 1.09) |
| Received treatment (PN) | 50.2 (162/323) | White  Mixed  Asian  Black  Other | 53.8 (134/249)  50.0 (3/6)  33.3 (18/54)  50.0 (5/10)  50.0 (2/4) | Reference  3.8  20.5  3.8  -- | Reference  96  80  96  -- | Reference  1.08 (0.48, 2.41)  **1.62 (1.09, 2.39)**  1.08 (0.57, 2.02)  -- |
|  | 49.5 (162/327) | Left full time education 19+ years old  <17  17-18 | 41.7 (70/168)  64.0 (48/75)  52.4 (44/84) | Reference  -22.3  -10.7 | Reference  122  111 | Reference  **0.65 (0.51, 0.83)**  0.80 (0.61, 1.04) |
|  | 49.5 (165/333) | 1- least deprived quintile of IMD  2  3  4  5 - most deprived | 52.8 (28/53)  45.2 (19/42)  60.3 (38/63)  45.6 (36/79)  45.8 (44/96) | Reference  7.6  -7.5  7.2  7.0 | Reference  92  108  93  93 | Reference  1.17 (0.77, 1.78)  0.88 (0.63, 1.21)  1.16 (0.82, 1.65)  1.15 (0.82, 1.61) |
|  | 49.1 (157/320) | Primaparous  Multiparous | 43.8 (60/137)  53.0 (97/183) | Reference  -9.2 | Reference  109 | Reference  0.83 (0.65, 1.04) |

Comparative statistics only computed where the denominator was greater than 5.
