# Supplementary material for: Inequalities in the identification and management of common mental disorders in the perinatal period: An equity focused re-analysis of a systematic review
Source: PLoS One. 2021 Mar 15;16(3):e0248631. doi: 10.1371/journal.pone.0248631 (PMC7959342; doi:10.1371/journal.pone.0248631)
Supplement: Attachment — Submitted filename: Response to reviewers R1.docx [file pone.0248631.s004.docx]

**We thank the reviewers and editor for the opportunity to revise our paper and respond to each point below.**

--------------------------------------------------------------------------------------------------------------

Journal Requirements:

When submitting your revision, we need you to address these additional requirements.

 1. Please ensure that your manuscript meets PLOS ONE's style requirements, including those for file naming. The PLOS ONE style templates can be found at

<https://journals.plos.org/plosone/s/file?id=wjVg/PLOSOne_formatting_sample_main_body.pdf> and

<https://journals.plos.org/plosone/s/file?id=ba62/PLOSOne_formatting_sample_title_authors_affiliations.pdf>

* **Thank you. We have restructured the title page and headings in the abstract (not track changes).**

2. Please include a copy of Table 5 which you refer to in your text on page 21.

* **Apologies, this was an error. The information is to be found in Supplementary Material Table 1, which is signposted. We have removed the words ‘(Table 5)’**

3. Please include captions for your Supporting Information files at the end of your manuscript, and update any in-text citations to match accordingly. Please see our Supporting Information guidelines for more information: http://journals.plos.org/plosone/s/supporting-information.

***We have split the supplementary file into S1 Table and S2 Table, renamed accordingly, and added captions at the end of the manuscript.**

[Note: HTML markup is below. Please do not edit.]

5. Review Comments to the Author

Please use the space provided to explain your answers to the questions above. You may also include additional comments for the author, including concerns about dual publication, research ethics, or publication ethics. (Please upload your review as an attachment if it exceeds 20,000 characters)

Reviewer #1: This is an innovative review that addresses an important question. While it is specific to the UK, I do think that there are implications for other jurisdictions as well. I have made some suggestions below.

*** Thank you for your review.**

Definition of the equity variables:
->In the methods equity variables should be more clearly defined. There is some discussion of this in the analysis section as it pertains to the quantitative papers, but should be expanded and include how they were defined for all papers.
->Why was parity considered as an equity variable?
->What groups were a priori considered advantaged vs. disadvantaged (as per Table 2)?
->For ethnicity, did you consider ethnicity and race (some of the categories seem to suggest so)?

*** We agree that our treatment of this was too brief and have added a new section, Categorisation of determinants, to the Methods (line 159), which details the distinction of advantaged from disadvantaged and provides information on why age and parity were included. The framework we have applied (PROGRESS-Plus) uses the word ‘race’ in the classification group that also includes ‘ethnicity’, ‘language’ and ‘culture’, and hence this is why the word appears in the paper. In the UK, self-ascribed ‘ethnicity’ and not ‘race’ is used in official statistics, including the census, which is the (rough) basis for measurement in research. By default, then, we were considering ‘ethnicity’ and not ‘race’, and have not used the word ‘race’ in the manuscript further than the definition. We think that the new section makes it more clear that the four concepts are simply grouped within this section as a whole, and it was not our intention to analyse by ‘race’. It remains a difficulty to remove it altogether, as the R in race forms part of the acronym PROGRESS (the framework is not UK-specific).**

Table 1 – Within column “Reported results by equity” consider defining how each of these variables was measured/defined in each study. E.g. self-reported ethnicity with XX categories.

*** Thank you, that is a logical progression from being more clear about classification in the Methods, and we have made these suggested changes in Table 1.**

Table 2
->The presentation of the results in the column “Summary of findings – effects (Q1)” is somewhat confusing. The referent group is not always clear. Sometimes the use of shorthand is a little too liberal. For example, for Khan 2015 and Russell/Lang 2013, not clear what “Ethnicity/language▼ Consultations using an interpreter, consultations with ethnic minority women” means.

*** Maintaining legibility while presenting this amount of complex information in Table 2 has been challenging. We are unable to see how we could completely overhaul it, so we have made the following changes which we think improves its readability. (1) We have moved the legend for ▲▼► to the column heading. (2) We have been more liberal with the descriptions for qualitative papers where the classification between advantaged and disadvantaged is not defined, and often in context to the interviewees’ narrative.**

Presentation of results generally
-> Headings should be consistently labeled (italics vs bolding etc) for clarity.

*** Thank you, we have corrected the erroneous heading levels.**

->“Absolute and Relative differences” are presented separately from the results above it – it might be better to integrate?

*** We did consider this when compiling the results, but we felt it important to keep the two separate; the former being results reported as per each paper, the latter being standardised calculations that we have performed. We have added a signpost under the heading ‘Absolute and relative differences’ (line 343) reminding the reader what this section represents.**

Other comments:
->“Paternalistic” is used in several places. Was this your interpretation that is was paternalistic, or labelled paternalistic in the study? If the latter, was this by the providers/patients or the study authors?

*** This was a descriptor used by the study authors to describe the approach taken by GPs for some women who seem reluctant to take ownership of their health. We agree that we have overused the word and not made its origins clear. To rectify, we have used quotes around it in Table 2, and also noted that it was described in the paper as such (line 413), and used other descriptors to describe the management approach taken by GPs (line 327).**

->How do your results fit into literature from other jurisdictions? How could lessons from your study inform practices in other countries?

*** We have added a sentence to ‘Implications for Policy and Practice’ in the Discussion (line 491) that outlines similar patterns in some other countries. We have also added that future research should include an international equity review (line 548).**

->Why was only one of the Prady papers (ref 22) included and not the other (ref 23)?

*** We included papers that were included in the NSC review. Reference 23 was excluded from the NSC review because “Women selected were diagnosed with mental health disorder prior to becoming pregnant” (Page 105, NSC review), i.e. the analysis was of an already selected sample.**

Reviewer #2: Thank you for this well-written and insightful paper. The authors have provided a unique contribution to the literature by applying a health equity lens to their re-review of UK-based studies examining detection and management of CMDs in the perinatal populations. A number of important barriers to equitable implementation of population-based programs are identified and well-considered suggestions for addressing these inequalities from research and practice perspectives are provided. Findings have applicability outside the UK.

*** Thank you for your review.**

The Methods and Results were clearly presented and led logically to the Discussion and Conclusion. The limitations of the study were also adequately addressed.

Minor suggestions for improvement:
1. Additional context around why migration (and also years since migration) specifically is an important factor to consider would be helpful

*** Thank you, it was our omission that we had not drawn this out. We have added a sentence in the discussion (line 502) outlining the consideration and highlighting the lack of evidence.**

2. Possible reasons for the observed differences in the magnitude of inequality among pregnant vs postnatal women would also be of interest

*** Due to the variation in the concepts measured between pregnant and postnatal women across (and within) studies, and the generally small sample sizes, we are unclear whether there are differences between the periods, and have added this as a limitation of the data in the discussion (line 458).**

3. Minor amendment: line 76, while the PHQ-2 and Whooley questions are similar they are not the same (for example, they ask about different intervals of time – PHQ-2 – last 2 weeks; Whooley questions – last month)

*** We have corrected this and cited Whooley et al (1997) paper in place of the PHQ-2 reference (line 76).**

I thank the authors again for this important work.
