# Supplementary material for: Inequalities in the identification and management of common mental disorders in the perinatal period: An equity focused re-analysis of a systematic review
Source: PLoS One. 2021 Mar 15;16(3):e0248631. doi: 10.1371/journal.pone.0248631 (PMC7959342; doi:10.1371/journal.pone.0248631)
Supplement: Attachment — Submitted filename: Prady Response to Reviewers R2.docx [file pone.0248631.s005.docx]

Dear Dr. Prady,

Thank you for submitting your manuscript to PLOS ONE. After careful consideration, we feel that it has merit but does not fully meet PLOS ONE’s publication criteria as it currently stands. Therefore, we invite you to submit a revised version of the manuscript that addresses the points raised during the review process.

==============================

The new additions to your manuscript were very helpful in understanding the framework you are applying.  One reviewer had additional comments along those lines which I think would help further clarify your conceptual approach

6. Review Comments to the Author

Please use the space provided to explain your answers to the questions above. You may also include additional comments for the author, including concerns about dual publication, research ethics, or publication ethics. (Please upload your review as an attachment if it exceeds 20,000 characters)

Reviewer #1: Thank you for addressing my comments. The edited sections are much clearer. Overall I think this is an interesting paper and addresses an important topic. I have just two remaining comments:
1) My main remaining question is regarding the rationale to include parity as an equity variable as it is more atypical, and age is already included which is the rationale given. Could you perhaps expand on your rationale or reference some prior work where this has been included as an equity variable? There are certainly ways parity I image parity could impact service use (e.g. multiparity making it harder to attend appointments if no childcare provided) but the rationale should just be more explicitly stated and referenced.
2) A limitation regarding the axes of inequity that were not under study (e.g. gender identity, sexual orientation, etc) could be considered.

**Thank you for your review, we are very grateful for your suggestions as the changes we have made have considerably strengthened our paper. Below we explain how we have addressed your remaining comments.**

**1) We agree it was not clear why we thought parity should be investigated, as what we had written equated it with age. We have amended this and added a sentence (page 8, line 168) to make our rationale clear about why we thought increased parity might be a disadvantaging factor in the UK context. We have added a notation in the Discussion indicating that inequity with increased parity has been noted in a non-UK study (page 29, line 494).**

**2). We have added a limitation relating to ‘our review’ (page 28 line 466) around the axes of inequity we did not apply as suggested.**
